# A Model for Risk Prediction of Cerebrovascular Disease Prevalence—Based on Community Residents Aged 40 and above in a City in China

**DOI:** 10.3390/ijerph18126584

**Published:** 2021-06-18

**Authors:** Qin Zhu, Die Luo, Xiaojun Zhou, Xianxu Cai, Qi Li, Yuanan Lu, Jiayan Chen

**Affiliations:** 1 Jiangxi Province Key Laboratory of Preventive Medicine, School of Public Health, Nanchang University, Nanchang 330006, China; 401437619004@email.ncu.edu.cn (Q.Z.); 401440318206@email.ncu.edu.cn (D.L.); zhouxiaojun@ncu.edu.cn (X.Z.); liqilq@ncu.edu.cn (Q.L.); 2 Center for Disease Control and Prevention of Qingyunpu District, Nanchang 330001, China; cxx0791@163.com; 3 Department of Public Health Sciences, University of Hawaii at Manoa, Honolulu, HI 96822, USA

**Keywords:** cerebrovascular disease, risk factors, risk prediction model, community residents

## Abstract

Cerebrovascular disease (CVD) is the leading cause of death in many countries including China. Early diagnosis and risk assessment represent one of effective approaches to reduce the CVD-related mortality. The purpose of this study was to understand the prevalence and influencing factors of cerebrovascular disease among community residents in Qingyunpu District, Nanchang City, Jiangxi Province, and to construct a model of cerebrovascular disease risk index suitable for local community residents. A stratified cluster sampling method was used to sample 2147 community residents aged 40 and above, and the prevalence of cerebrovascular diseases and possible risk factors were investigated. It was found that the prevalence of cerebrovascular disease among local residents was 4.5%. Poisson regression analysis found that old age, lack of exercise, hypertension, diabetes, smoking, and family history of cerebrovascular disease are the main risk factors for local cerebrovascular disease. The relative risk ORs were 3.284, 2.306, 2.510, 3.194, 1.949, 2.315, respectively. For these six selected risk factors, a cerebrovascular disease risk prediction model was established using the Harvard Cancer Index method. The R value of the risk prediction model was 1.80 (sensitivity 81.8%, specificity 47.0%), which was able to well predict the risk of cerebrovascular disease among local residents. This provides a scientific basis for the further development of local cerebrovascular disease prevention and control work.

## 1. Introduction

Cerebrovascular disease mainly refers to the abnormal manifestations of the nervous system caused by cerebral vascular hemorrhage and infarction ischemia. It is characterized by sudden, dangerous, difficult-to-treat, and irreversible sequelae. It is the main disease that endangers the health and quality of life of middle-aged and elderly people [1]. The report shows that cerebrovascular disease is the leading cause of death globally and is also the main cause of disability in adults [2]. Cerebrovascular disease has become one of the major global public health problems because of its high morbidity, disability, and mortality [3]. Numerous studies have demonstrated that high blood pressure [4,5,6], diabetes [4,7], dyslipidemia [8], smoking [9] and other bad behaviors and lifestyles are recognized risk factors for cerebrovascular disease. Practice has proved that health education, interventions for high-risk behaviors, and active treatment of hypertension and other related diseases can effectively control the occurrence of cerebrovascular disease, reduce its complications, and improve quality of life [10]. Studies have revealed a presence of low public awareness and low control rate regarding risk factors for cerebrovascular disease [11], which seriously affects the acceptance and cooperation of cerebrovascular disease intervention, and then affects its intervention effect. For this reason, researchers from all over the world, combined with local actual conditions, collected relevant risk factors and used different mathematical models to predict the occurrence and death of cerebrovascular diseases [12,13,14,15,16,17,18]. Such effort will allow us to obtain accurate figures such as relative risk, disease or death risk, etc., which will provide important support for the intervention of cerebrovascular disease. It has become more clear today that predicting the risk of cerebrovascular disease is of great significance.

When many scholars predict the occurrence and death risks of cerebrovascular disease, they are combined with the local reality to make predictions, mainly because the absolute risk of cerebrovascular disease varies from race to race [19]. In addition, the risk of cerebrovascular disease assessed by risk assessment tools may not be suitable for other regions [20]. This provides support for us to carry out the risk prediction of cerebral blood pressure in the Qingyunpu District of Nanchang City, Jiangxi Province. Some researchers set up logistic regression [21] and COX regression [22] predictive models. Since the characteristics of the low incidence of cerebrovascular disease and the nature of chronic non-communicable diseases are more suitable in the Poisson distribution, this study employed a Poisson regression model to explore the risk factors and relative risk of cerebrovascular disease. Some studies have suggested that the Harvard Cancer Index risk method can better predict the risk of chronic diseases and tumors [23,24]. In this study, community residents over 40 years old in Qingyunpu District of Nanchang City were used as the research object, and the Poisson regression model was used to explore the risk factors and relative risks that affect the characteristics of cerebrovascular disease in local residents. The Harvard Cancer Index risk method was used to convert the relative risk into a risk score to predict the risk of cerebrovascular disease of local residents. The establishment of the model system help local primary health service agencies screen out high-risk groups of cerebrovascular disease that meets the actual local conditions and establish local conditions. In addition, it will provide a scientific basis for the establishment of comprehensive prevention and treatment measures for cerebrovascular diseases with local characteristics.

## 2. Materials and Methods

### 2.1. Participants

Nanchang City has six districts and three counties under its jurisdiction. Among them, Qingyunpu is the central city of Nanchang and an important industrial zone of the area. The former large state-owned enterprises in Nanchang City were all located in this area. Due to the basic transformation of enterprise, the residents in the area are mostly employees and their families. The unique characteristics of community residents provide a specific basis for this research.

The participants in this study were community residents aged 40 years old and above that were living in Nanchang, Jiangxi Province, China. The inclusion criteria were: (1) inhabitants who have lived in the area for more than half a year at the time of the survey, (2) being aged 40 and above, and (3) willing to participate in the study and sign the informed consent form. The exclusion criteria were: (1) not being a resident, and (2) those who have not lived in the area for more than half a year.

### 2.2. Sampling Methods

The stratified cluster sampling method was used in this study. One district in the central city of Nanchang, Jiangxi Province, was selected. Two towns were randomly selected from six towns in the district, and one community was selected from each of the two selected towns. All community residents who met the inclusion criteria in the sampled communities were recruited in the survey. A total of 2147 participants were enrolled in this study. Nine logically unqualified cases were removed from the samples following data reviewing and double-checking. The final sample enrolled in this study was 2138 cases, and the overall effective rate was 99.6%.

### 2.3. Survey Content and Method

The questionnaire used in this study was adapted from the template uniformly developed by the Jiangxi Provincial Center for Disease Control and Prevention. The questionnaire’s content includes (1) demographics such as gender, age, marital status, occupation, and education level; (2) lifestyle such as exercise habits, smoking, and alcohol drinking; (3) family history of cerebrovascular disease; (4) past medical history such as cerebrovascular disease, hypertension, and diabetes; (5) physical examination such as height, weight, systolic blood pressure (SBP), diastolic blood pressure (DBP), and pulse; (6) laboratory tests, which include fasting blood glucose, glycosylated hemoglobin, blood cholesterol, triglycerides, high-density lipoprotein, and low-density lipoprotein. This household survey was conducted from April 2018 to December 2018. The investigation was organized by the Qingyunpu Center for Disease Control and Prevention and conducted by trained general practitioners and general nurses in a community health service center. Laboratory tests were carried out by the laboratory of the local community health service center.

### 2.4. Criteria

Hypertension refers to the following instances: (1) During the investigation, the respondents reported that they were previously diagnosed by a clinician and had taken antihypertensive drugs in the on-site inspection. Regardless of the current blood pressure level, they were counted as hypertensive patients. For those who had no history of taking antihypertensive drugs, the blood pressure result measured this time was used as the diagnosis basis. (2) During the investigation, if the respondent has not been diagnosed by a clinician, the result of the judgment of hypertension should prevail in this study. The diagnostic criteria are as follows: the SBP ≥ 140mmHg and/or the DBP ≥ 90 mmHg [25]. Dyslipidemia means one or more of the following were detected: the total cholesterol ≥ 6.22 mmol/L (240 mg/dL), triglycerides ≥ 2.26 mmol/L (200 mg/dL), high-density lipoprotein < 1.04 mmol/L (40 mg/dL), or those who are currently taking lipid-lowering drugs [26]. Diabetes refers to fasting plasma glucose ≥ 7.0mmol/L or 2-h postprandial plasma glucose ≥ 11.1mmol/L, or people who are currently taking hypoglycemic agents or insulin [27]. Type of stroke was categorized into four groups: intracerebral hemorrhage, subarachnoid hemorrhage, ischemic stroke, and undetermined stroke [28]. Transient ischemic attack refers to a sudden, focal neurological deficit due to vascular cause, lasting <24 h [28].

In the present study, age was grouped as 40–59 and ≥60 years because the number of illnesses in China was relatively high in these two age groups [29]. Smoking/smokers were defined as those who smoke continuously or accumulatively for ≥6 months in their lifetime. Alcohol drinking was defined as the consumption of alcohol every day for more than one year. The frequency of alcohol drinking was classified into three groups: never drink, drink occasionally, and drink frequently (liquor, ≥3 times per week and ≥100 mL each time). Type of physical exercise was categorized into two groups: sufficient physical exercise (taking physical exercise ≥3 times a week and ≥30 min of moderate intensity exercise each time or engage in moderate or heavy physical labor) and lack of physical exercise. Obesity was defined as body mass index ≥ 28 kg/m^2^.

### 2.5. Grouping

The samples were randomly divided into a modelling group (*n* = 1069) and a validation group (*n* = 1069). We adopted a lottery method to stipulate the allocation of odd and even numbers to the participants. The participants with odd numbers were allocated to the modelling group (i.e., 1, 3, 5, 7, 9, …, 1137), and those with even numbers were allocated to the validation group (i.e., 2, 4, 6, 8, 10, …, 1138). The modelling group was used for establishing the risk prediction model, while the validation group was used to validate the model.

### 2.6. Quality Control

First, the questionnaire and survey plan were developed on the basis of the template formulated by the Provincial Center for Disease Control and Prevention, which is widely used and validated in Chinese settings. The second is the sampling process. Residents were sampled using a stratified cluster sampling method. The modeling group and the verification group were randomly divided into two groups according to the rules of odd and even numbers. These processes were used to ensure the representativeness of the sample and control the bias of non-processing factors between the groups on the results. Third, all the staff participating in the on-site investigation were medical staff from the local community health service center. They had a medical background and received unified training. Fourth, in the process of organization and implementation, the research team took advantage of the local health administrative department to promote the project’s smooth development through health administrative means. Simultaneously, the research team also designated professionals to supervise and review the on-site investigation process. Fifth, double-entry and logical review were both carried out during the data analysis stage.

### 2.7. Statistical Analysis

Counting data was expressed by rate or composition ratio, and *χ*^2^ test was used for comparison between groups. The trend analysis used the trend *χ*^2^ test. The Wilcoxon rank sum test was used for comparison of the risk level of two groups. The risk prediction model was developed by the Poisson regression model. The area under the receiver operating characteristic (ROC) curve (AUC), sensitivity and specificity, and other indicators were used to evaluate the predictive effect of the model. The significance level was set at two-tailed *α* = 0.05.

The risk factor model was constructed using a Poisson regression model. Poisson distribution is often used to describe the random distribution of the total number of rare particles in unit time, unit plane, or unit space. Since the prevalence of cerebrovascular diseases in this study is relatively low, and the prevalence data are discrete distribution data, it particularly meets the requirements of the Poisson regression model.

### 2.8. Calculation of Individual Risk Level of Cerebrovascular Disease

Table 1 and Table 2 show the conversion standard of risk scores and risk levels based on the Harvard Risk Index [23,24] and the Colditz’s standard [23,30]. The calculation of the risk level of individual cerebrovascular disease was conducted as follows:

(1) The odds ratio (OR) of each risk factor screened by the Poisson regression model was converted into the risk scores of each impact factor according to the conversion standard shown in Table 1. For example, there are n risk factors that affect the cerebrovascular system, which are recorded as f_1_, f_2_, f_3_…f_n_. Poisson regression model can be used to calculate the relative risk OR of each risk factor, which are OR_f1_, OR_f2_, OR_f3_…OR_fn_. According to the range of OR value in Table 1, the corresponding risk score (RC) can be found. The risk scores of each risk factor were RC_f1_, RC_f2_, RC_f3_…RC_fn_.
(2) Calculate the average risk score of the population. First of all, on the basis of the factors f_1_, f_2_, f_3_…f_n_ that affect cerebrovascular diseases screened by the Poisson regression, consult relevant literature and find the exposure rate (ER) of each influencing factor in the population. The exposure rates of each risk factor were recorded as ER_f1_, ER_f2_, ER_f3_…ER_fn_. Then, the average risk score (average risk score of the population, AROP) was calculated with the risk score value and exposure rate of the influencing factors.
  In our study, the AROP was calculated through the following means:
  (1) According to the China health statistics yearbook 2019 [31], the exposure rate (ER) of CVD patients aged 60 or above in China in 2018 was 0.17.
  (2) According to Chinese guidelines for the prevention and treatment of hypertension (revised edition 2018) [32], the ER of CVD patients who had a medical history of hypertension in China in 2017 was 0.28.
  (3) According to Guidelines for the Prevention and Treatment of Type 2 Diabetes in China (2017 edition) [33], the ER of CVD patients who had a medical history of type 2 diabetes in China in 2016 was 0.11.
  (4) According to a Chinese report on stroke prevention and treatment 2017 [34], the ER of CVD patients with a medical history of stroke in China in 2016 was 0.28.
  The specific method for calculation:$\;AROP=\overset{i}{\underset{n=1}{\sum }}ER_{fi}*RC_{fi}$
(3) Calculation of the risk score of individual (RCI): Refer to the scoring standard in Table 1, and according to the questionnaire answered by the individual, the risk score points were accumulated if there was a risk factor; otherwise, the risk score was recorded as 0. The individual risk factor scores were RCI_f1_, RCI_f2_, RCI_f3_…RCI_fn_. Sum the risk scores of each risk factor formed the total risk value of the individual.
  The specific calculation method is: $RCI=\overset{i}{\underset{n=1}{\sum }}RCI_{fi}$
(4) Calculation of the risk of individual cerebrovascular disease *R* is as follows:
  The specific calculation method is: $R=\frac{\overset{i}{\underset{n=1}{\sum }}RCI_{fi}}{\overset{i}{\underset{n=1}{\sum }}ER_{fi}*RC_{fi}}$.

According to the disease risk level table in the risk assessment model of the Harvard Cancer Index method (Table 2), we divided the individual cerebrovascular risk level R into different levels.

## 3. Results

### 3.1. The Prevalence of Cerebrovascular Disease

This study showed a cerebrovascular disease prevalence of 4.5% among the participants. As shown in Table 3, the higher the age, the higher the disease’s prevalence rate of the disease. The prevalence was significantly higher among males (6.3%), smokers (8.4%), and the participants who lack exercise (8.0%), with hypertension (6.6%), diabetes (10.5%), and a family history of the disease (7.9%).

### 3.2. The Risk Factors of Cerebrovascular Disease

The Poisson regression analysis showed that age, exercise habits, hypertension, diabetes, smoking, and family history of cerebrovascular disease were related to the participants’ cerebrovascular disease (Table 4).

### 3.3. Development of the Risk Prediction Model

The Poisson regression results of this study (Table 4) were used to determine the risk factors’ OR size. This section refers to Table 1 to convert various risk factors into specific risk scores. The risk scores of age and diabetes were both 25 points, and the other risk factors were all 10 points (Table 5). The average risk score of the participants of modelling group was determined to be 22.20 in this study. 

### 3.4. Validation of the Risk Prediction Model

As shown in Table 6, the prevalence of cerebrovascular disease increased with the risk level in the modelling group, the validation group for predicting risk level, and the validation group of actual risk level (*χ*^2^*_trend_* = 22.583, *p* < 0.001; *χ*^2^*_trend_* = 21.149, *p* < 0.001, *χ*^2^*_trend_* = 16.144, *p* < 0.001, respectively), indicating that the classification of risk level is reasonable. Wilcoxon rank sum test showed that there was no significant difference in the proportion of risk level between the modeling group and the validation group for predicting risk level (*z* = 0.895, *p* = 0.371). It was also no significant difference in the proportion of risk level between the validation group for predicting risk level and the validation group of actual risk level (*z* = 1.124, *p* = 0.261). This shows that the effect of the risk prediction model is stable.

The ratio *R* was obtained by substituting the validation group’s data into the developed risk prediction model. As shown in Table 7, the *R* ratio for predicting the total population’s risk was 1.80 (sensitivity = 81.8%, specificity = 47.0%), which was considered the optimal positive cut-off point to predict the onset of cerebrovascular disease. The optimal positive cut-off point for the males and the females were 2.03 (sensitivity = 53.9%, specificity = 69.6%) and 1.58 (sensitivity = 72.2%, specificity = 56.5%).

**Figure 1:**
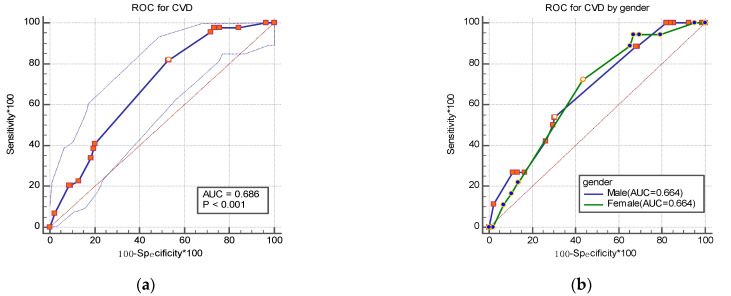

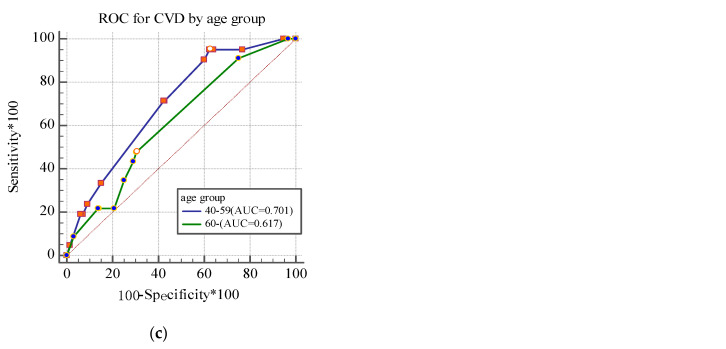
Receiver operating characteristic (ROC) curve for identifying cerebrovascular disease by gender (**a**,**b**) and age group (**c**) in the study.

As shown in Figure 1a, the AUC for identifying cerebrovascular disease was 0.686 (95%*CI*: 0.657~0.714) in the total sample. The AUCs were 0.664 (95%*CI*: 0.617~0.709) in men and 0.66 (95%*CI*: 0.626~0.700) in women (*p* = 0.990) (Figure 1b). The AUCs of the age group were 0.701 (95%*CI*: 0.666~0.734) in the 40–59 age group and 0.617 (95%*CI*: 0.564~0.668) in the ≥60 age group (*p* = 0.250) (Figure 1c).

## 4. Discussion

Previous studies have shown that hypertension, hyperlipidemia, lack of exercise, and diabetes are risk factors for cardiovascular disease [35,36]. Similar findings were found in this study showing that age, hypertension, diabetes, lack of exercise, smoking, and family history of cerebrovascular disease were risk factors for cerebrovascular disease. Thus, the timely detection of these risk factors and interventions is of great significance to preventing and controlling cerebrovascular diseases. 

This study found that older ages were associated with a higher prevalence of the CVD. Roughead et al. [37] described a similar finding in that the older the age, the higher the hospitalization rate. This may be related to the physiology of the elderly. The complexity and severity of the disease will increase with age, which will lead to the increased utilization of inpatient health services.

This study found that hypertension and diabetes were significant risk factors for cerebrovascular disease, consistent with previous studies [38,39,40]. Previous studies have demonstrated that about 54% of strokes and 47% of ischemic heart disease worldwide are due to hypertension [38,39]. The incidence and mortality of cerebrovascular disease in diabetic patients are significantly higher than those in non-diabetic patients [40]. Diabetes can cause the basement membrane of tiny blood vessels to thicken, atherosclerosis of small and medium blood vessels, and large and medium blood vessels, leading to secondary hypertension and dyslipidemia, which are the pathological basis of ischemic stroke.

This study showed that smokers are 1.949 times more likely to suffer from cerebrovascular disease (95%CI: 1.015~3.534) than non-smokers. Some reports have shown that both active and passive smoking can increase the occurrence of atherosclerosis [41]. Pirie et al. [42]. Reported that the risk of stroke decreases with the increase of time to quit smoking, showing that smoking cessation is essential to preventing cerebrovascular disease.

This study shows that people who lack physical exercise have a higher risk of cerebrovascular disease (95%CI: 1.258~4.068) compared with people who regularly exercise. Large cohort studies confirmed that lack of exercise could increase the morbidity and mortality of the cardiovascular disease and stroke risk [43,44]. It may be because physical activity can effectively regulate blood lipids and blood pressure, thereby reducing the risk of cerebrovascular disease. These results indicate that the community should actively call on adults to exercise. This study also found that the OR of people with a family history of cerebrovascular disease was 2.315 (95%CI: 1.167~4.593), higher than those with no family history of stroke. Other studies have demonstrated that a family history of stroke is a significant risk factor for stroke onset [45,46,47]. The influence of family history on risk factors may go through two links [48]: (a) the genetic susceptibility to stroke risk factors such as hypertension increases and (b) family eating habits may affect their relatives or offspring.

The primary purpose of this study was to develop a risk prediction model for cerebrovascular disease. We found six risk factors (age, exercise habits, hypertension, diabetes, smoking, and family history of cerebrovascular disease) related to cerebrovascular disease. Thus, a risk prediction model was initially developed. This model was tested in the present study and showed excellent validity in detecting cerebrovascular disease.

The results showed that the AUC for identifying the cerebrovascular disease in the community residents was 0.686, and 0.664 in both men and women. AUC values were all between 0.5 and 0.7, indicating that the model has a reasonable degree of discrimination. The ratio *R* of the model for total sample was 1.80 (sensitivity = 81.8%, specificity = 47.0%), which was determined to be the optimal positive cut-off point to predict the onset of the cerebrovascular disease. The sensitivity for the total population in this study was 81.8%, which is higher than the 53.0% reported from a recent study, indicating potentially clinical value of the model.

This study has several limitations. First, this research data came only from one district in Jiangxi Province, China, which may be suitable only for the sub-cultural background of the local area. The sample size needs to be further increased to improve the representative and accuracy of the model. Second, the cerebrovascular disease risk prediction model constructed in this study was based on a cross-sectional study. The optimization of the model requires further follow-up validation in community populations. Third, the multi-factor model of this study did not include other risk factors for cerebrovascular diseases, such as dyslipidemia. The risk prediction model also lacks specific general applicability in application and promotion due to differences in regional economic conditions and eating habits. Fourth, the male and female positive cut-off values of the cerebrovascular disease model constructed in this study are quite different. 

## 5. Conclusions

In summary, this study showed that the prevalence of cerebrovascular disease among community residents over 40 years old in Nanchang City, Jiangxi Province, was 4.5%, which provided basic data on cerebrovascular disease in this area. In addition, this study also found that the important risk factors for local cerebrovascular diseases of local residents including old age, lack of exercise, high blood pressure, diabetes, smoking, and family history of cerebrovascular disease with the relative risk ORs of 3.284, 2.306, 2.510, 3.194, 1.949, and 2.315, respectively. The cerebrovascular disease risk prediction model established on the basis of these six risk factors had an *R* value of 1.80 (sensitivity 81.8%, specificity 47.0%), which can be used to better predict the risk of cerebrovascular disease of local residents. Findings from this study may provide a scientific basis for the further development of local cerebrovascular disease prevention and control strategies. At the same time, the findings from this study may form a basis for the future need for more large-scale research and verification in terms of the Chinese population.

## Figures and Tables

**Table 1:** Conversion standard of the cerebrovascular disease risk score.

Odds Ratio (OR)	Strength of Association	Mark	Risk Score
0.9~1.1	Not significant		0
0.7~0.9 or 1.1~1.4	Weak	− or +	5
0.4~0.6 or 1.5~2.9	Moderate	−− or ++	10
0.2~0.3 or 3.0~6.9	Strong	−−− or +++	25
<0.2 or 7.0~	Very strong	−−−− or ++++	50

**Table 2:** Classification of individual cerebrovascular disease risk levels.

R	Risk Levels
R < 0	Very low
0 < R < 0.5	Lower
0.5 < R < 0.9	Low
0.9 < R < 1.1	Moderate
1.1 < R < 2.0	High
2.0 < R < 5.0	Higher
R > 5.0	Very high

**Table 3:** The prevalence of cerebrovascular disease.

		Modelling Group		Validation Group		Total		
		*n*	%	*n*	%	*n*	Prevalence of CVD	*p*
Gender	Male	401	37.51	421	39.38	822	6.3	0.001
	Female	668	62.49	648	60.62	1316	3.3	
Age	40–59	299	27.97	284	26.57	583	3.1	<0.001
	60-	770	72.03	785	73.43	1555	7.2	
Physical exercise	Lack of exercise	188	17.59	201	18.80	389	8.0	<0.001
	Exercise regularly	881	82.41	868	81.20	1749	3.7	
Smoking	No	911	85.22	907	84.85	1818	3.8	<0.001
	Yes	158	14.78	162	15.15	320	8.4	
Alcohol drinking	No	927	86.72	907	84.85	1834	4.7	0.275
	Yes	142	13.28	162	15.15	304	3.3	
Obesity	No	839	78.48	830	77.64	1669	4.4	0.624
	Yes	230	21.52	239	22.36	469	4.9	
Hypertension	No	488	45.65	467	43.69	955	1.9	<0.001
	Yes	581	54.35	602	56.31	1183	6.6	
Dyslipidemia	No	601	56.22	614	57.44	1215	4.0	0.167
	Yes	468	43.78	455	42.56	923	5.2	
Diabetes	No	920	86.06	913	85.41	1833	3.5	<0.001
	Yes	149	13.94	156	14.59	305	10.5	
Cardiopathy	No	940	87.9	934	87.37	1874	4.3	0.318
	Yes	129	12.07	135	12.63	264	5.7	
Family history of CVD	No	925	86.53	911	85.22	1836	3.9	0.002
	Yes	144	13.47	158	14.78	302	7.9	
Total		1069		1069		2138	4.5	

**Table 4:** The risk factors of cerebrovascular disease.

	β	Wald χ^2^	*p*	OR ^#^	95%CI
Constant	–5.405	90.808	<0.001		
Age group					
40–59				1	
60-	1.189	4.046	0.025	3.284	1.310~11.019
Physical exercise					
Exercise regularly				1	
Lack of exercise	0.836	7.897	0.005	2.306	1.258~4.068
Smoking					
No				1	
Yes	0.667	4.469	0.035	1.949	1.015~3.534
Hypertension					
No				1	
Yes	0.920	6.607	0.010	2.510	1.296~5.351
Diabetes					
No				1	
Yes	1.161	16.138	<0.001	3.194	1.786~5.586
Family history of CVD					
No				1	
Yes	0.839	5.768	0.016	2.315	1.167~4.593

^#^ OR, odds ratio.

**Table 5:** Development of the risk score of cerebrovascular disease.

Risk Factors	OR ^#^	Risk Score	Exposure of the Reference Population	
			Size	Resource
Age (≥60)	3.171	25	0.17	China health statistics yearbook 2019
Physical exercise	2.306	10	0.82	Data from this study
Hypertension	2.436	10	0.28	Chinese guidelines for the prevention and treatment of hypertension (revised edition 2018)
Diabetes	3.220	25	0.11	Guidelines for the Prevention and Treatment of Type 2 Diabetes in China (2017 Edition)
Smoking	1.877	10	0.28	Chinese report on stroke prevention and treatment 2017
Family history of CVD *	2.315	10	0.14	Data from this study

^#^ OR, odds ratio. * CVD, cerebrovascular disease.

**Table 6:** The risk levels and the prevalence of the two groups.

Risk Level	Modeling Group		Validation Group (Predicted Risk Level)		Validation Group (Actual Risk Level)	
	N Enrolled (%)	N Illness (%)	N Enrolled (%)	N Illness (%)	N Enrolled (%)	N Illness (%)
Very low	30 (2.8)	0 (0.0)	35 (3.3)	0 (0.0)	0 (0.0)	0 (0.0)
Relatively low	123 (11.51)	0 (0.0)	126 (11.8)	0 (0.0)	0 (0.0)	0 (0.0)
Low	0 (0.0)	0 (0.0)	0 (0.0)	0 (0.0)	0 (0.0)	0 (0.0)
General	75 (7.0)	2 (2.6)	88 (8.2)	1 (0.8)	307 (30.0)	4 (1.3)
Relatively high	283 (26.5)	11 (3.7)	241 (22.5)	7 (2.9)	234 (22.8.)	5 (2.1)
High	506 (47.3)	39 (7.2)	579 (54.2)	36 (6.2)	484 (47.2)	35 (6.7)
Total	1069 (100.0)	52 (4.9)	1069 (100.0)	44 (4.1)	1069 (100.0)	44 (4.1)

**Table 7:** Sensitivity, specificity, Youden index, positive predictive value (PPV), and negative predictive value (NPV) of the model cut-off points in identifying cerebrovascular disease in men and women in this study.

Cut-Off Point	Male					Female					Total				
	Sensitivity%	Specificity%	Youden index	PPV%	NPV%	Sensitivity%	Specificity%	Youden index	PPV%	NPV%	Sensitivity%	Specificity%	Youden Index	PPV%	NPV%
0.45	100.0	7.6	0.076	6.6	100	94.4	20.6	0.151	3.3	99.1	97.7	15.6	0.133	4.7	99.4
0.90	100.0	14.4	0.144	7.1	100	94.4	30.3	0.248	3.7	99.4	97.7	24.2	0.219	5.2	99.6
1.13	100.0	16.0	0.160	7.3	100	94.4	33.2	0.276	3.9	99.4	97.7	26.5	0.243	5.4	99.6
1.35	100.0	17.7	0.177	7.4	100	88.9	34.6	0.235	3.7	98.9	95.5	28.1	0.236	5.4	99.3
1.58	88.5	31.4	0.199	7.8	97.6	72.2	56.5	0.287	4.5	98.6	81.8	46.8	0.287	6.2	98.4
1.80	88.5	31.9	0.204	7.9	97.7	-	-	-	-	-	81.8	47.0	0.288	6.2	98.4
2.03	53.9	69.6	0.235	10.4	95.8	22.2	86.2	0.084	4.4	97	40.9	79.8	0.207	7.7	96.9
2.25	50.0	70.1	0.201	9.9	95.1	22.2	86.5	0.087	4.5	97	38.6	80.2	0.188	7.4	96.8
2.48	42.3	73.7	0.160	9.6	94.5	16.7	89.4	0.060	4.3	96.9	34.1	81.6	0.157	7.0	96.6
2.70	26.9	83.3	0.102	11.9	94.8	11.1	93.0	0.041	4.3	96.8	20.5	90.6	0.111	8.6	96.3
3.15	11.5	97.7	0.093	25	93.8	0.0	97.9	−0.021	0.0	96.6	6.8	97.9	0.047	12.0	95.9

## Data Availability

Data are made available by contacting the corresponding authors.

## Abbreviations

AUC	Area under the curve
CVD	Cerebrovascular disease
DBP	Diastolic blood pressure
OR	Odds ratio
ROC	Receiver operating characteristic
SBP	Systolic blood pressure
